# Supplementary material for: A double ovulation protocol for Xenopus laevis produces doubled fertilisation yield and moderately transiently elevated corticosterone levels without loss of egg quality
Source: PLoS One. 2024 Jul 19;19(7):e0299179. doi: 10.1371/journal.pone.0299179 (PMC11259257; doi:10.1371/journal.pone.0299179)
Supplement: Attachment — Submitted filename: Response to reviewers.docx [file pone.0299179.s002.docx]

**PONE-D-24-05117 Moss et al.**

**Response to Reviewers**

We thank the reviewers for their thoughtful comments and have replied to these in detail to improve the paper.

The full text of “Review Comments to Author” are reproduced here with our responses in blue.

**Reviewer #1:**

This article describes the use of a double ovulation procedure for Xenopus as opposed to the traditional single ovulation procedure. Overall the results are sound and show that a second quicker induced ovulation can work with Xenopus. However there are a few questions raised by the study that need to be addressed.

1) The corticosterone levels are raised by the second ovulation, similar to those in shipped frogs. Is this something a lab would want to put the frogs through on a regular basis? Since frogs can live for 20+ years what are the long term effects?

New text has been added to the Discussion section to address this question and recognise that it is relevant and open (lines 265-269).

2) No mention of age of frogs or whether they were virgins. This should be addressed.

More complete details of the frogs, including that they were virgins and their age are now given in the Methods section (lines 314-315)

3) Seven frogs were removed from the trial and perished. Why? This is not common for Xenopus laevis.

Text has been added to explain that six frogs were lost due to drowning in a tank overflow malfunction and only one was lost because of an infection, which is not unreasonable for a Xenopus colony over a 21 month period where there are multiple (including sometimes inexperienced) personnel involved (324-325).

4) Was feeding increased between ovulations?

No – but feeding was always ad libitum, i.e. excess chow was provided and leftovers removed after 30-40 minutes. Text describing this has been added to the Methods section (lines 345-346).

5) Does amount of hormone change egg output? Would the use of PMSG before hCG change?

We did not investigate this alternative, which would require its own systematic investigation. (As numbers of eggs laid are usually excess to requirements, the inclusion of an additional injection to further increase numbers was not an obvious question for us to ask.)

6) Seems that the rate of in vitro fertilizations was low across the board. Why did the researchers not simply squeeze the eggs out as most labs do instead of collecting in 1X MBS? Normally with X. laevis labs achieve near 100% fertlization.

We chose not use the “squeeze” method for expressing eggs from the females but instead left them to lay their eggs in a solution that maintains fertilisability to minimise handling stress and maximise consistency of eggs for fertilisation. Text explaining this reasoning has been added to the text (lines 354-357).

**Reviewer #2:** Moss and colleagues propose a double ovulation protocol for Xenopus leavis as opposed to the single ovulation protocol currently used as standard frog fertilisation protocol. Traditionally the laboratory protocol for obtaining Xenopus eggs is to induce one round of ovulation and egg laying, and then to wait 3 months before anther induction. The rationale behind the double ovulation approach is that in the wild, frogs undergo successive matings within the mating seasons. Therefore, it may be expected that two rounds in rapid succession of ovulation prior to the prolong interval would be possible without harming the animals, and would double egg yield. The authors state that the egg laying capacity of Xenopus leavis is potentially higher than observed during the standard procedure, and a double ovulation would result in a higher fertilisation yield, a reduction in animal use in accordance with the 3Rs (Replacement, Reduction and Refinement) and benefits in cost-savings. The authors thoroughly test egg yield, egg quality, and stress hormones in the mother from this approach and find that the double ovulation approach indeed increases effective egg yield with acceptable perturbation to the mother. With minor changes to the text and figures, the protocol described by Moss and colleagues represents a valuable contribution to the scientific community using Xenopus leavis as a model organism.

Minor points:

The authors compared a double ovulation protocol to the single ovulation standard protocol over a time period of 21 months and evaluated the egg number, fertilisation rate, and stress response (corticosterone levels) of the animals during the ovulation cycles of the study. However, there is no reference given for the standard protocol or a reference to a published protocol on which the author's standard protocol is based (line 65).

Three references that specify the current “standard of care” rest period have been added (lines 68-69).

In the standard ovulation protocol described in this paper, female frogs are injected with chorionic gonadotropin, thereby stimulating ovulation followed by egg laying. After ovulation, frogs rest for a 3-month period before the procedure is repeated. In the proposed double ovulation protocol, female frogs are re-injected seven days after the first ovulation, and the 3-month resting period starts after the second ovulation. This procedure was repeated for seven 3-month cycles in total, and data was presented for ovulation cycles 1, 2, 6 and 7. Although it is sufficient to show data from cycles 1, 2 and 6,7 it is not clear why data from cycles 3, 4 and 5 are not shown. It would have been interesting to see the extent of variability throughout the trial.

In retrospect, we would also have liked to have had data for those cycles, but in fact, due to personnel and procedure changes in our animal facility during the period in question beyond our control, the variability (in both groups) was certainly higher then and so not comparable with the cycles we do report on.

The arrangements of the plots, consistency in labelling and references to the figures in the text and legends of figures 1–3 need to be checked carefully and corrected accordingly. The plots in the figures lack labelling, such as A,B, C, etc. However, in the text, references to Fig.x A,B are given. For more details, see “additional points” below.

Figures and text have now been amended to include panel labels and references to them in the text have been checked throughout (lines 121, 149, 187).

Data in Figures 1, 2 and 3 are shown in two different representations within the figure. On one hand, with bar plots, and on the other hand, with box plots. It is not clear from the text why this representation was chosen. For simplicity and a better understanding of the data, I recommend skipping the bar plots and using box plots only. Moreover, I suggest combining plots from Figure 1 and Figure 2 into one figure. In this way, plots representing egg count can be directly compared to the fertilisation rate.

Figures 1 and 2 have been combined as suggested. We have kept the histogram plots because they make the trends much easier to see, while the box-and-whisker plots reveal and emphasise the variability. We have added text in the figure captions to explain the reason for re-plotting.

In the discussion, the authors strongly focus on the limitations of the study. I recommend to slightly shift the focus to the findings and data rather than the variabilites or missed opportunities in experimental measurements (lines 244 -247). It is absolutely valid to point out the limitations of the study, but it is important to put these limitations into context with the presented data and point out the positive aspects of the findings. This has been done for most of the paragraphs of the discussion but is missing in paragraphs 244-247. I would suggest re-formulating this paragraph. In line 244, it says, “Second, some further measurements that could have been informative were not made.” The question “Why?” immediately comes to mind. Give arguments, why the shown data is still representative and enough to support the study.

We thank the reviewer for these encouraging words and have amended the Discussion to be more positive (lines 246-249, 250, 262-269).

Overall, the data presented in this study supports the advantages of the double ovulation protocol over the single ovulation protocol proposed by the authors. Despite variabilites introduced during the trial, such as changes in animal care staff, feeding changes, and technical issues in the animal facility, the authors applied appropriate statistical testing and could show that the double ovulation protocol resulted in a higher egg yield without loss of egg quality compared to the standard single ovulation protocol. Although elevated levels of stress were experienced by the frogs during the second ovulation protocol, these levels were only transient and fall within an acceptable range for Xenopus leavis.

Additional points:

• Figure 1 and Figure 2: I would suggest merging Figure 1 and Figure 2 and showing only the box plots. I recommend labelling the plots in the figure properly for better reference in the text and figure legends (e.g., A,B). Figure legends need to be updated accordingly.

Figures 1 and 2 have been merged as suggested to make a new Figure 1, but we have retained the histogram plots as panels in the figure as these are simply easier to read and will save anyone who wants an at-a-glance version of the data from having to re-plot it from the raw numbers.

• Figure 1, right panel, top plot: Here, the x-axis is labelled “ovulation group”. The other plots have the x-axis labelled “frog ovulation group”. I strongly suggest labelling the x-axis consistently across plots.

Corrected

• Figure 2 and Figure 3, the two top plots have their x-axis labelled “ovulation group” and the bottom plots with “frog ovulation group”. Again, I strongly suggest labelling plots consistently.

Corrected

• Figure 3: The y-axis of the plots in figure 3 is labelled “Corticosterone level” but no unit is given, such as [pg/hr].

Corrected

• Line 59: The citation given here is a bit confusing, as it is not consistent with the citation format within the rest of the manuscript, and it is not clear if, where, and when the survey has been published.

The numbers reported were from an informal survey carried out to help inform the development of the Xenbase.org community web page. The text has been amended to clarify that this is unpublished data.

• Line 142: Here, a reference is given to Figure 2 “Fig.2A”. However, no “A” is assigned to a plot in the figure.

Corrected

• Line 182, there is a reference to “Fig. 3A,B”. However, there is no “A” or “B” assigned to the plots presented in figure 3.

Corrected

• Line 227: The sentence ends with an open exclamation mark. Is the sentence in the figure legend complete?

The legend was missing a full stop, which has now been added.

• Line 286: There is no information given on the age of the frogs. Were the freshly purchased frogs the same age? Was the age well-balanced between the different groups?

More details about the frogs, including their age and age homogeneity have been added (lines 314-315).
