# Supplementary material for: A double ovulation protocol for Xenopus laevis produces doubled fertilisation yield and moderately transiently elevated corticosterone levels without loss of egg quality
Source: PLoS One. 2024 Jul 19;19(7):e0299179. doi: 10.1371/journal.pone.0299179 (PMC11259257; doi:10.1371/journal.pone.0299179)
Supplement: Attachment — Submitted filename: Response to reviewers May 2024.docx [file pone.0299179.s003.docx]

**PONE-D-24-05117R1 Moss et al.**

**A double ovulation protocol for Xenopus laevis produces doubled fertilisation yield and moderately transiently elevated corticosterone levels without loss of egg quality**

**Response to reviewers**

Reviewer #1: The authors have addressed all the comments although i do not agree with their response to point #6. They stated that they did not squeeze the frogs to lay eggs because they wanted to maximise consistency of eggs for fertilization, but then in same sentence later they state that they get lower fertilization this way.

We have have re-written the section (lines 334-338) to explain more carefully that our method sacrificed yield to achieve more consistency and that this was our priority for a side-by-side study.

Reviewer #2: Moss et al. have adequately addressed and implemented the suggested comments. Besides a few comments on text formatting, the manuscript is acceptable for publication.

- In the revised manuscript showing tracked changes, a different title is given, however the revised manuscript without tracked changes has the original title.

We apologize for this discrepancy. The re-submission versions now all have the same title.

- Figure 1 C/D: y-axis label in C shows “Fertilization Rate %”, y-axis label in D shows “Fertilization Rate (%)”. Add the parentheses in C.

Corrected.

- Figure 1 G: It looks like the y-axis label has a bigger font size than the other axis labels in this figure. Adjust font size for consistency.

Corrected.

- 205-206: In the legend of Figure 2 the reference to A, B, C, D is bold. However, in all other figure legends letters are not bold. Make them consistent.

Corrected.

- 227-230: Here, the parenthesis at the end of the sentence is open “…significant difference between the groups (two-tailed paired t-test…”.

Corrected.

- 262: “It would indeed be interesting to track differences the recovery time for corticosterone levels after ovulation, but for this study…”. Check the sentence – it looks like a word is missing between “differences” and “the recovery time”.

Corrected, and the sentence also split into two to improve readability.
